# The Emergence of a New Isoform of POU2F1 in Primates through the Use of Egoistic Mobile Genetic Elements

**DOI:** 10.1134/S1607672922020107

**Published:** 2022-05-10

**Authors:** B. M. Lyanova, A. P. Kotnova, A. A. Makarova, Yu. V. Ilyin, S. G. Georgieva, A. G. Stepchenko, E. V. Pankratova

**Affiliations:** Engelhardt Institute of Molecular Biology, Russian Academy of Sciences, Moscow, Russia

**Keywords:** *POU2F1* gene, mobile genetic elements, head carcinoma

## Abstract

The emergence of new genes and functions is of paramount importance in the emergence of new animal species. For example, the insertion of the mobile element Tigger 2 into the sequence of the functional gene *POU2F1* in primates led to the formation of a new chimeric primate-specific isoform POU2F1Z, the translation of which is activated under cellular stress. Its mRNA was found in all species of monkeys, starting with macaques. Analysis of the fragments of the Tigger2 copy corresponding to the human exon Z showed that the splicing sites of exon Z are homologous in humans and in most monkeys, with the exception of lemurs and galagos. The stop codon introduced into the mRNA by the Tigger2 sequence is present in all primates, starting with macaques. The internal ATG codon is also present in all primates, with the exception of lemurs and galagos. In the course of evolution, other MGEs, mainly of the SINE type, were inserted into the Tigger2 copy. In the course of evolution, both the location and the number of mobile SINE elements within the *POU2F1* gene changed. Starting with macaques, the pattern of the arrangement of SINE elements within the Tigger2 copy in the studied region of the *POU2F1* gene was fixed and then remained unchanged in other primates and humans, which may indicate its functional significance.

POU2F1 is overexpressed in many cancers and tumor cell lines. A high level of the POU2F1 protein correlates with an extremely unfavorable prognosis for the course of the disease [1–7]. For example, Sharpe et al. [3] showed an increased level of POU2F1 expression in head and neck carcinoma cell lines compared to non-cancer cell samples of the same tissues from the same patients. *POU2F1* knockdown resulted in a significant decrease in proliferation and growth in cell lines of these tumor types. POU2F1 was shown to regulate regeneration and tumor transformation in the HCT116 cell line (rectal tumor cells) [5] and in other tumors [4, 6–9].

Interestingly, the level of the detected POU2F protein often does not correlate with the level of the corresponding mRNA. For example, a high content of POU2F1 mRNA was found in the bone marrow, but the amount of the corresponding protein in this tissue is insignificant. In the rectum, conversely, the amount of the corresponding mRNA is relatively small, whereas the amount of the protein is one of the highest. This is possible if the POU2F1 expression is regulated to a large extent at the level of translation.

We have discovered a new POU2F1Z isoform whose translation increases many times under cellular stress. One of the functions of this isoform is to protect cells from the damaging effects of various types of stress [10]. POU2F1Z mRNA is expressed in all human tissues and cells, but it is found at the maximum level in the cerebral cortex and in lymphoid cells. We detected a high level of POU2F1Z protein expression in Burkitt’s lymphoma Namalwa tumor cells. Interestingly, two mRNAs were found in humans (MT294127 and AK091438.1), from which the same POU2F1Z protein is translated (UniProt KBP14859-3). These mRNAs are transcribed from two alternative promoters of the *POU2F1* gene and differ in 5'-end sequences. POU2F1Z contains an additional Z exon located between exons 3 and 4. During splicing, this exon introduces a stop codon into the mRNA sequence, followed by an ATG codon coinciding with the “main” reading frame of the POU2F1 protein. For the resulting mRNA, the POU2F1Z isoform with a new N-terminal domain is synthesized from this ATG triplet, and a short peptide is synthesized from the ATG codon located in the first exon (Fig. 1).

**Fig. 1.**
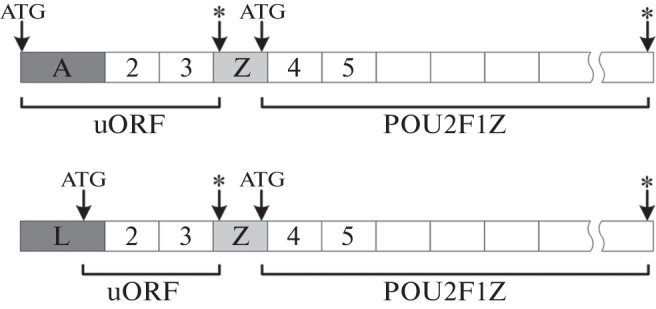
Structural scheme of two mRNA isoforms POU2F1 from which the same POU2F1Z protein is translated. Exon Z is formed from the Tigger2 transposon sequence. uORF is a short open reading frame at the 5' end of an mRNA whose translation starts at ATG codons of alternative exons A(U) or L. Asterisks indicate the stop codons. The start of translation of the POU2F1Z isoform is indicated with ATG in the exon Z region.

Under normal conditions, the synthesis of this short peptide (uORF) inhibits the translation of the long reading frame (POU2F1Z protein); however, under cellular stress, the synthesis of the short peptide is suppressed and the translation of the POU2F1Z protein is activated [10].

We have previously shown that activation of POU2F1Z translation starts under the action of damaging agents and protects cells from cell death [10]. We assume that this defense mechanism can significantly increase the resistance of cancer cells to chemotherapeutic drugs and non-drug methods of treating malignant tumors.

In this work, we investigated the emergence of the POU2F1Z isoform in the course of evolution and also performed a search for and comparative analysis of this isoform in animals of the subtype Craniata.

As part of the study of the origin of the Z exon, we aligned its sequence with the genomes of various mammals and found in the chromosomes of different species many insertion sites for the sequences that have significant homology with exon Z. We assumed that exon Z might represent a transposable element and, in the next step, ran its sequence through the transposable element database at NCBI GeneBank. We found that exon Z, from which the *POU2F1Z*  isoform is transcribed, is a fragment of a copy of the Tigger2 DNA transposon inserted into the *POU2F1* gene between exons 3 and 4 and corresponds to the 2150–2461 bp region of this transposon (Fig. 2).

**Fig. 2.**
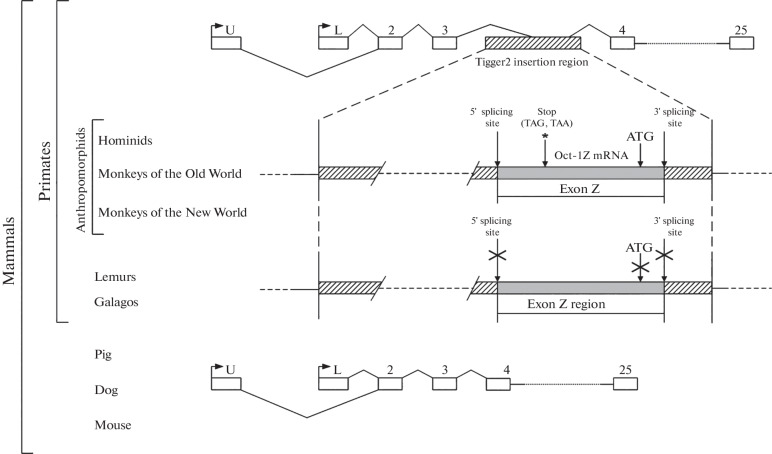
Tigger2 transposon fragment inserted into the *POU2F1* gene. Exons are indicated with rectangles. Arrows indicate the transcription starts. The lines indicate the splicing sites. In all primates, the Tigger2 fragment has a deletion (indicated by the dotted line). In all primates except lemurs and galagos, the Tigger2 fragment contains splicing sites, a stop codon, and an ATG codon. In lemurs and galagos, despite the presence of the Tigger2 fragment in the *POU2F1* gene, the formation of the POU2F1Z mRNA isoform is impossible, similarly to other mammals that lack the Tigger2 transposon in the *POU2F1* gene.

Tigger2 is an ancient DNA transposon found in the genomes of mammals, including humans. The length of the complete reconstructed variant is 2718 bp. Includes transposase gene (from 603 to 2483 bp) flanked by 24 bp inverted repeats. The complete variant of Tigger 2 has not been found in mammalian genomes; however, its various fragments are found everywhere [11].

Next, we performed a study to determine at what point in the evolutionary development of animals of the subtype Craniata Tigger 2 was integrated into the *POU2F1* gene.

For this purpose, we analyzed the sequences of the *POU2F1* orthologues of animals of the Craniata subtype contained in the NCBI database. In total, we analyzed 279 species. A hidden Markov model was obtained for the Tigger2 transposon from the Dfam database [12]. HMMER (ver. 3.3.2) [13] was used to search for Tigger2 fragments in *POU2F1* sequences retrieved from NCBI. For this analysis, we considered it necessary to use the search method based on hidden Markov models, which is implemented in HMMER, since it was shown to be more sensitive (compared to blastn and other methods based on the search for homology with a single sequence) in the search for fragments of ancient transposons [14].

Files with annotations of the reference genomes of the species included in our sample were retrieved from the NCBI database. Fragments corresponding to *POU2F1* orthologs were extracted from the retrieved annotations. The resulting annotations, as well as the HMMER results, were superimposed on the corresponding reference gene sequences.

We found that the POU2F1Z isoform is primate-specific and is absent in other animal species. In addition, unlike other primates, Tigger2 was not found in the tarsier *POU2F1* gene.

We showed that, in all primate *POU2F1* genes studied, the inserted copy of Tigger2 is divided into several parts and has a deletion in the region 1341 (–44)–2119 bp. In the *POU2F1* genes of lemurs and galagos, the deleted Tigger2 copy is divided into three parts, whereas in the *POU2F1* genes of other primates it is divided into four parts. It is known that the coding region of the Tigger2 transposon is located in the region 603–2483 bp, which means that the Tigger2 regions that are inserted in the gene cannot encode a complete transposase but can retain the properties of its individual domains.

Today, databases for all primates contain only simulated versions of *POU2F1* mRNA sequences and proteins translated from them. However, according to modeling data, mRNA and protein of the POU2F1Z isoform are already expressed in macaques, the lowest representatives of the Old World monkeys.

Next, to determine whether of mRNA and protein of the POU2F1Z isoform is expressed in primates, we analyzed the region of the Tigger2 copy inserted into the primate *POU2F1* gene and corresponding to the human Z exon.

When analyzing the splicing sites of the human Z exon, we found that, among monkeys, the corresponding sequences are completely homologous to the sequences of the splicing sites of the human exon Z, with the exception of nocturnal monkeys, in which there is one nucleotide substitution in the acceptor site of the putative Z exon. In lemurs and galagos, there are significant differences from the splicing sites of the human Z exon, and it can be assumed that, despite the presence of the Tigger2 fragment in this region of the gene, this region does not get in mRNA, because alternative splicing of this region is impossible (Fig. 2).

We also analyzed the sequences of the putative stop codon and ATG codon in the studied region of the inserted Tigger2 copy for correspondence with those in the human Z exon. It turned out that the TAG stop codons in this region are completely homologous for all primates with the exception of macaques, in which the putative corresponding stop codon is TAA (Fig. 2).

The corresponding translation start points (ATG codon) are present in all primates except for lemurs and galagos (Fig. 2), which have CTC and GTG triplets, respectively, instead of the ATG codon.

To understand what events caused the splitting of the Tigger2 copy within the *POU2F1* gene into several parts, using the Dfam database of mobile elements, a region of the *POU2F1* gene in primates with an inserted copy of the Tigger2 transposon was analyzed. As a result, we found that other mobile genetic elements, mainly of the SINE type, were inserted into the Tigger2 copy. In the course of evolution, both the location and the number of mobile SINE elements within the study area changed. These changes occurred in the studied *POU2F1* genes of lower primates up to macaques. The location of mobile SINE genetic elements, established in the studied region of the *POU2F1* gene in macaques, remains unchanged in other primates (Old World monkeys and humans), which may indicate its functional significance.

It can be assumed that it was at this stage of evolution that the 2150–2461-bp region of Tigger2 got into the coding sequence of the *POU2F1* gene, which entailed positive evolutionary changes and, as a result, led to the exon Z formation. 

Our results show that the insertion of the mobile element became part of the functional primate *POU2F1* gene through a stepwise process that included transposition, loss of the ability to move further due to insertion of other mobile elements into Tigger2, and subsequent transcriptional and translational fusion with the target gene. In our study, we showed that a new chimeric primate-specific POU2F1Z isoform resulted from the fusion of the *POU2F1* transcription factor gene and the Tigger 2 transposase gene fragment, which gave rise to a new exon of the primate *POU2F1* gene.

Emergence of new genes and functions is of primary importance for the formation of new animal species. At present, various types of duplications in the formation of new genes are well studied [15]. The creation of new genes through the use of material from selfish mobile genetic elements is much less understood. Analysis of such evolutionary events is a difficult task because it is often the result of small sequence substitutions or structural rearrangements whose functional significance is difficult to determine.

In the case of the *POU2F1* gene in humans and primates, this transformation led to the emergence of the new POU2F1Z isoform, which protects cells from the damaging effects of cellular stress but creates difficulties for the treatment of malignant neoplasms.
